# Effective Dose of Ramosetron for Prophylaxis of Postoperative Nausea and Vomiting in High-Risk Patients

**DOI:** 10.1155/2015/951474

**Published:** 2015-07-14

**Authors:** Seongheon Lee, Sinho Jeong, Joungmin Kim, Seongwook Jeong

**Affiliations:** Department of Anesthesiology and Pain Medicine, Chonnam National University Medical School, Gwangju 519-763, Republic of Korea

## Abstract

*Background*. Postoperative nausea and vomiting (PONV) are common adverse events with an incidence of up to 80% in high-risk patients. Ramosetron, a selective 5-HT_3_ receptor antagonist, is widely used to prevent PONV. The purpose of this study was to evaluate the effective dose of ramosetron for the prevention of PONV in high-risk patients. *Methods*. Fifty-one patients were randomly allocated to 3 groups and were administered ramosetron 0.3 mg (group A), 0.45 mg (group B), or 0.6 mg (group C), at the end of their surgery. The episodes of PONV were assessed 1, 6, 24, and 48 hours after the injection and all the adverse events were observed. *Results*. The complete response rate in the postoperative period 6–24 hours after the anesthesia was higher in group C than in group A: 93% versus 44%. Group C's experience score of Rhodes index was lower than group A's: 0.81 ± 2.56 versus 3.94 ± 5.25. No adverse drug reaction could be observed in all groups. *Conclusions*. The effective dose of ramosetron to be injected for the near-complete prophylaxis of PONV 6 to 24 hours after surgery in high-risk patients is a 0.6 mg bolus injection at the end of the surgery.

## 1. Introduction

Postoperative nausea and vomiting (PONV) are very common and distressing adverse events after general anesthesia. The general incidence is about 30% to 50%, and the PONV rate can go up to 80% in subsets of high-risk patients [1, 2]. It has been reported that PONV may prolong the recovery period and hospital stay, causing high patient dissatisfaction [3]. It is easier to prevent than to treat after occurrence, and anesthesiologists therefore have to find a strategy to attenuate the incidence of PONV and patient-related distress in order to reduce the physical and economic expenses [4–7].

Several antiemetics of different pharmacological classes are available to prevent PONV in patients scheduled to receive elective surgery. These include anticholinergics [8, 9], antihistamines [10], phenothiazines [11], and butyrophenones [12]. Currently, selective 5-hydroxytryptamine type 3 (5-HT_3_) receptor antagonists (ondansetron, granisetron, tropisetron, and dolasetron) are frequently used in the prevention of PONV, due to their efficacy and lesser adverse effects than other antiemetics [13–15].

Ramosetron (ramosetron hydrochloride), a selective 5-HT_3_ receptor antagonist, has been on the market as an antiemetic drug for cancer patients receiving chemotherapy [16, 17] or anesthesia [18–20] in Japan and a number of other Asian countries since 1996. It has been reported that the administration of a single dose of intravenous ramosetron 0.3 mg is sufficient for prophylaxis following general anesthesia [21]. In the case of high-risk patients [1], however, there is no recommended protocol to control PONV.

The Rhodes index of nausea, vomiting, and retching (RINVR) is an instrument composed of eight 5-point self-reported items designed to assess the subjective and objective factors of nausea, vomiting, and retching in various situations, including in surgical patients [22]. The RINVR is a more suitable method for the quantitative and objective assessment of the degree of PONV felt by patients than the simple detection of the PONV occurrence or the subjective measurement of the degree of PONV using a visual analogue scale (VAS). The reliability and validity of the Korean translated version of the RINVR has already been established [23].

As little is known about the effective ramosetron dose for the prevention of PONV without inducing undesirable adverse effects such as severe headache, dizziness, or drowsiness in high-risk patients, this study was conducted as a prospective, randomized, and double-blinded study to determine the effective dose of ramosetron for the prevention of PONV in high-risk patients undergoing elective surgery, using the RINVR and the rate of emesis-free patients as assessment tools.

## 2. Methods

### 2.1. Study Design

This study was designed as a prospective, randomized, double-blind, parallel-armed single-center trial. It was approved by the Institutional Review Board of the Chonnam National University Hwasun Hospital (Hwasun, Republic of Korea), and the written informed consent of all patients was obtained. A total of 51 American Society of Anesthesiologist Physical Status (ASA PS) I or II patients aged over 19 years with a score of 2–4 on the Apfel's PONV risk scale [1] and scheduled to undergo elective surgery under general anesthesia were allocated to receive ramosetron 0.3 mg (*n* = 17, group A), ramosetron 0.45 mg (*n* = 17, group B), or ramosetron 0.6 mg (*n* = 17, group C). They were randomly allocated into 3 groups by a computerized randomization table. The code of the assigned group was delivered to the anesthesiologist within the opaque envelope from the principal investigator. Patients were excluded from the study if they presented a known allergy to ramosetron, anemia, active hepatitis, or a history of blood donation over 450 mL in the 30 days prior to the surgery. Patients who had been medicated with antiemetics, steroids, or antihistamines within 24 hours of the surgery were also excluded. The primary endpoint of the study was to find the minimal effective dose of ramosetron for the complete prevention of postoperative nausea and vomiting.

According to previous studies, the incidence of PONV in high-risk patients is estimated as 80% [1, 2]. We set the primary end point to 40% of PONV. Using type I (*α* = 0.05, two-sided test) and II errors (*β* = 0.2, power = 0.8), the sample size was estimated at 15 for each group and was increased to 17 considering a dropout rate of 10%.

All patients were made to fast from midnight on the day of the surgery, and no premedication was administered before the surgery. The patients were monitored with ECG, pulse oximetry, end-tidal carbon dioxide, invasive arterial pressure (Datex-Ohmeda S/5, Planar Systems, Inc., Beaverton, OR, USA), and Bispectral Index System (BIS, Aspect 2000, Aspect Medical Systems, Inc., Newton, MA, USA) monitors. Before the anesthesia, a venous catheter was inserted and an infusion of lactated Ringer's solution was initiated at a rate of 10 mL/kg/h. Following full preoxygenation with 100% oxygen, anesthesia was induced with 2.0 mg/kg of intravenous propofol and a 2 ng/mL remifentanil effect-site targeted infusion, followed by rocuronium 0.8 mg/kg. When neuromuscular block was achieved, the trachea was intubated and the anesthesia was maintained with sevoflurane and a target-controlled infusion (TCI) of remifentanil to prevent signs of inadequate anesthesia.

At the end of the surgery, the investigated drug, ramosetron (Nasea, Astellas Pharma Inc., Tokyo, Japan), was administered according to the patient group, and the exact time was recorded. To guarantee a double-blind trial, an independent research nurse prepared the investigational drugs with a 10 mL dilution. The patients were randomly allocated to each group and were administered an intravenous injection of 0.3 mg of ramosetron (group A), 0.45 mg of ramosetron (group B), or 0.6 mg of ramosetron (group C). The neuromuscular block was antagonized by the administration of pyridostigmine and glycopyrrolate.

After full recovery from the anesthesia in the PACU (Post-Anesthetic Care Unit), the patients received intravenous patient-controlled analgesia (IV-PCA) for 2 days. The IV-PCA device (Accufuser plus, Wooyoung Medical Co. Ltd, Seoul, Republic of Korea) contained fentanyl 1500 *μ*g and ketorolac 180 mg in 100 mL saline (1 mL of basal infusion rate and 1 mL of patient-controlled dose in a 15-minute lockout time).

### 2.2. Efficacy Measures

All episodes of PONV (nausea, vomiting, and retching) and the severity of the PONV were assessed by an independent research nurse 1, 6, 24, and 48 hours after injection of the investigational drug, using the Rhodes index (Table 1). According to the literature [24], nausea was defined as the subjectively unpleasant sensation associated with the awareness of the urge to vomit; retching was defined as the labored, spastic, rhythmic contraction of the respiratory muscles without expulsion of the gastric contents; and vomiting was defined as the forceful expulsion of gastric contents from the mouth. At the end of each observation period, the patients evaluated the severity of their PONV using the RINVR [22]. A complete response (i.e., emesis-free) was defined as no PONV and no need for another rescue antiemetic medication. Rescue antiemetics were administered for active nausea and vomiting, and the first-line rescue treatment consisted of intravenous metoclopramide 10 mg. If the patient did not respond to the initial treatment within 15 minutes, it was followed by an intravenous injection of 1 mg of ondansetron as a second-line treatment, with a maximum of 5 administrations. The need for rescue antiemetic and analgesic agents was also recorded.

### 2.3. Safety Profile Measures

The safety profiles of the different concentrations of ramosetron were evaluated based on the incidence rates of adverse events, the vital signs, and a clinical laboratory test (complete blood cell count, urinalysis and alkaline phosphatase, aspartate aminotransferase (AST), alanine aminotransferase (ALT), blood urea nitrogen, creatinine levels, and coagulation parameters (prothrombin time, activated partial thromboplastin time, and platelet count)). All adverse events were assessed.

### 2.4. Statistical Analysis

All statistical analyses were performed using the SPSS software (version21.0; SPSS Inc., Chicago, IL, USA). A logistic regression analysis and a Pearson *χ* ^2^ test were performed. A *p* value below 0.05 was considered statistically significant. All the data were expressed as mean (SD) ± standard deviation (SD) or proportions of patients (%).

## 3. Results

### 3.1. Study Patients Characteristics

Fifty-one patients were enrolled in this study, and four patients (1 patient from group A, 1 patient from group B, and 2 patients from group C) were dropped from the study for violating the protocol. There were no significant differences in the age, body weight, height, and physical status of the three groups (Table 2). In addition, the Apfel PONV risk scores (female gender, nonsmoking status, history of PONV and/or motion sickness, and postoperative opioid were attributed 1 point each) did not differ between the groups.

### 3.2. Efficacy of Ramosetron in Dose Escalation

The complete response rate in the postoperative period 6–24 hours after anesthesia was higher in group C than in group A: 93% (*n* = 14) versus 44% (*n* = 7). Group C's experience score of Rhodes index was lower than group A's: 0.81 ± 2.56 versus 3.94 ± 5.25 (Table 3). There were no significant differences in the complete response rates and Rhodes index scores in the other observed periods. The number of patients in need of rescue antiemetics was as follows: 3 subjects in group A, 5 in group B, and 2 in group C, without statistical significance.

### 3.3. Safety Profile Analyses

None of the subjects complained of severe headache, dizziness, or drowsiness, which are known as common adverse effects from the administration of 5-HT_3_ receptor antagonists. Several patients experienced fever and chills, and one patient suffered from hypertension. These symptoms were not considered to be related to the drug (Table 4). There was no serious QTc prolongation or withdrawal caused by adverse events. No clinically significant laboratory findings were observed in the examination 7 days after the surgery.

## 4. Discussion

Numerous reports and practice guidelines have been published about the prophylaxis of postoperative nausea and vomiting [25, 26]. However, it remains one of the most unfavorable possible complications after general anesthesia, particularly in high-risk groups [26]. This problem is multifactorial in origin, and influential factors include the age, obesity, history of motion sickness and/or previous PONV, menstrual cycle, surgical procedure, anesthetic technique, and postoperative pain [24].

Currently, 5-HT_3_ antagonists are the most widely-used agents for the prevention of PONV. Despite the well-known shared mechanism of action of these drugs, they have their own distinguished chemical structures and demonstrate variable receptor binding affinities, durations of action, and dose responses. Ramosetron has been proven to have a high affinity with 5-HT_3_ receptors and a long plasma half-life, which causes a long duration of action and strong potency [27]. Unlike with other 5-HT_3_ antagonists, the ramosetron manufacturer sets the recommended dose for PONV at the same level as for CINV (chemotherapy induced nausea and vomiting). The author could not easily understand how a dosage of 0.3 mg had been decided for PONV, while a maximal dose of 0.6 mg is used in the clinical trials for drug development.

Unfortunately, very few studies have supported the effects of ramosetron on the prevention of PONV. Although Fujii and Tanaka published many results from clinical trials with ramosetron [19], most of these were fabricated [28]. In an article, Carlisle commented that “the data showed such unusual distributions as to suggest that sampling had not been random and that, therefore, the data should be excluded from any meta-analysis [29].” About 15 of Fujii et al.'s articles have been retracted, and more proof is therefore needed for the effect of ramosetron on the prophylaxis of PONV [28].

The efficacy of intravenous ramosetron for the prevention of PONV after laparoscopic cholecystectomy and gynecological surgery has previously been evaluated without sorting the patients according to their risk level. Ryu et al. [20] reported that an intravenous bolus of ramosetron 0.3 mg was as effective as ondansetron 8 mg for PONV prophylaxis after laparoscopic cholecystectomy. Kim et al. [21] reported that intravenous 0.3 mg ramosetron was as effective as intravenous 8 mg ondansetron for decreasing the incidence of PONV and reducing the severity of nausea in female patients in the first 24 hours following gynecological surgery. Additionally, Ryu et al. [30] reported that the combination of 0.1 mg oral and 0.3 mg intravenous ramosetron was more effective than either 0.3 mg intravenous ramosetron or 0.1 mg oral ramosetron only for the prophylaxis of nausea and vomiting in the first 24 hours after laparoscopic cholecystectomy surgery. They emphasized the importance of a multifarious approach to PONV.

This study only included Apfel's high-risk patient group [1]. The men's score was 2 or 3, and the women's score was 3 or 4. Although they presented a high risk of PONV, the incidences of nausea, vomiting, and retching were not higher than in the existing data or in our expectations. Despite the published data, we considered that a 0.3 mg dose of intravenous ramosetron would be sufficient to attenuate the incidence of PONV [1, 2] as we wanted to eradicate PONV in the high-risk group without causing any adverse events. Moreover, we needed a more objective assessment tool for the degree of PONV. We selected the Rhodes index of nausea, vomiting, and retching (RINVR), as the reliability and validity of its Korean translated version has been confirmed [23].

Our results demonstrated that the effect of ramosetron on the prevention of PONV in high-risk patients undergoing major laparoscopic surgery is only dose-dependent in the 6–24-hour period following the operation. In the other time intervals, a dose-dependent reaction was only observed without statistical significance. Despite the use of appropriate assessment tools for PONV, we must recognize that nausea and vomiting are highly irregular and idiopathic symptoms.

Ramosetron lacks the sedative, dystrophic, and extrapyramidal symptoms associated with other non-5-HT_3_ antiemetics, such as droperidol and metoclopramide [24]. A number of previous studies have shown that ramosetron is relatively free of adverse effects and can be safely used in the control of PONV after major surgery. No adverse drug reaction, including headache, dizziness, drowsiness, or QTc prolongation, was observed in the three groups involved in this clinical trial.

The study presented several limitations. First, for ethical reasons, we failed to enroll a placebo group of patients. Second, we could not distinguish the sexual difference in the randomization process. According to the Apfel PONV risk scale, being female is one of the risk factors, and the highest risk factor score for male volunteers was therefore 3. The recruitment and analysis should have been performed differently. Third, we could not conclude that ramosetron 0.6 mg is completely safe. The sample size of 5~16 patients for each group is definitively too small to draw any significant conclusions for the adverse events. More than 500 patients per group in large phase-IV trials would be necessary. These limitations call for further studies.

In conclusion, intravenous ramosetron 0.6 mg appears to be a more effective dose than ramosetron 0.3 mg for the prophylaxis of PONV in high-risk patients in the 6–24-hour period after major laparoscopic surgery.

## Figures and Tables

**Table 1:** Rhodes index of nausea, vomiting, and retching (RINVR).

The Rhodes index of nausea, vomiting, and retching (RINVR)					
(1) In the last () hours, I threw up () times.	7 or more (4)	5-6 (3)	3-4 (2)	1-2 (1)	I did not throw up (0)

(2) In the last () hours, from retching or dry heaves, I have felt () distress.	No (0)	Mild (1)	Moderate (2)	Great (3)	Severe (4)

(3) In the last () hours, from vomiting or throwing up, I have felt () distress.	Severe (4)	Great (3)	Moderate (2)	Mild (1)	No (0)

(4) In the last () hours, I have felt nauseated or sick at my stomach ().	Not at all (0)	1 hour or less (1)	2-3 hours (2)	4–6 hours (3)	More than 6 hours (4)

(5) In the last () hours, from nausea/sickness at my stomach, I have felt () distress.	No (0)	Mild (1)	Moderate (2)	Great (3)	Severe (4)

(6) In the last () hours, each time I threw up I produced a () amount.	Very large (3 cups or more) (4)	Large (2-3 cups) (3)	Moderate (1/2–2 cups) (2)	Small (up to 1/2 a cup) (1)	I did not throw up (0)

(7) In the last () hours, I have felt nauseated or sick at my stomach () times.	7 or more (4)	5-6 (3)	3-4 (2)	1-2 (1)	No (0)

(8) In the last () hours, I have had periods of retching or dry heaves without bringing anything up () times.	No (0)	1-2 (1)	3-4 (2)	5-6 (3)	7 or more (4)

Total experience scores: sum of all scores, total occurrence score: 1 + 4 + 6 + 7 + 8, total distress score: 2 + 3 + 5.

**Table 2:** Patient characteristics.

	Group A (*N* = 16)	Group B (*N* = 16)	Group C (*N* = 15)
Age (years)	60.6 ± 10.5	57.0 ± 11.3	60.9 ± 17.6
Sex (male : female)	5 : 11	3 : 13	4 : 11
Height (cm)	161.3 ± 6.5	156.6 ± 7.0	159.9 ± 6.5
Weight (kg)	59.9 ± 8.7	55.4 ± 8.4	63.7 ± 9.7
ASA PS status	1.56 ± 0.51	1.63 ± 0.5	1.73 ± 0.5
Apfel's PONV risk [1]	3.31 ± 0.95	3.38 ± 0.81	3.00 ± 0.85

The values are shown as means ± SD or numbers of patients.

ASA PS: American Society of Anesthesiology Physical Status.

PONV: Postoperative nausea and vomiting.

**Table 3:** Rhodes index of nausea, vomiting, and retching (RINVR) and incidence of complete responses.

	Group A (*N* = 16)	Group B (*N* = 16)	Group C (*N* = 15)
During 0~1 hr			
Complete responses	12 (75%)	9 (56%)	13 (87%)
Occurrence score	0.71 ± 1.16	0.82 ± 1.01	0.41 ± 0.94
Distress score	0.82 ± 1.81	0.71 ± 0.99	0.35 ± 0.86
Experience score	1.53 ± 2.81	1.53 ± 1.94	0.81 ± 1.80

During 1~6 hrs			
Complete responses	13 (81%)	10 (63%)	12 (80%)
Occurrence score	0.76 ± 1.92	1.00 ± 1.58	0.53 ± 1.23
Distress score	0.65 ± 1.97	0.65 ± 1.11	0.29 ± 0.69
Experience score	1.50 ± 3.92	1.80 ± 2.72	0.88 ± 1.96

During 6~24 hrs			
Complete responses	7 (44%)	13 (81%)	14 (93%)^*∗*^
Occurrence score	2.12 ± 2.74	1.18 ± 3.09	0.53 ± 1.74
Distress score	1.59 ± 2.50	0.94 ± 2.77	0.24 ± 0.75
Experience score	3.94 ± 5.25	2.25 ± 5.98	0.81 ± 2.56^*∗*^

During 24~48 hrs			
Complete responses	14 (88%)	13 (81%)	14 (93%)
Occurrence score	0.47 ± 1.33	0.65 ± 1.54	0.47 ± 1.50
Distress score	0.35 ± 1.22	0.65 ± 1.66	0.29 ± 0.99
Experience score	0.88 ± 2.50	1.38 ± 3.26	0.81 ± 2.56

^*∗*^ *p* < 0.05 as compared with group A.

The values are shown as means ± SD or proportions of patients (%).

**Table 4:** Adverse events.

	Group A (*N* = 16)	Group B (*N* = 16)	Group C (*N* = 15)
Fever & chills	4 (25%)	3 (19%)	3 (20%)
Hypertension	1 (6%)	0	0
Severe headache	0	0	0
Dizziness	0	0	0
Drowsiness	0	0	0
Significant changes of laboratory test	0	0	0

The values are shown as the number of patients (proportion of patients %).
